# Comparative study of cytotoxic effects induced by environmental genotoxins using XPC- and CSB-deficient human lymphoblastoid TK6 cells

**DOI:** 10.1186/s41021-019-0130-y

**Published:** 2019-07-16

**Authors:** Akira Sassa, Takayuki Fukuda, Akiko Ukai, Maki Nakamura, Michihito Takabe, Takeji Takamura-Enya, Masamitsu Honma, Manabu Yasui

**Affiliations:** 1 Department of Biology, Graduate School of Science, Chiba University, Chiba, 263-8522 Japan; 2 Tokyo Laboratory, BoZo Research Center Inc, 1-3-11, Hanegi, Setagaya-ku, Tokyo, 156-0042 Japan; 3 Division of Genetics and Mutagenesis, National Institute of Health Sciences, 3-25-26 Tonomachi, Kawasaki-ku, Kawasaki, 210-9501 Japan; 4 Department of Chemistry, Kanagawa Institute of Technology, 1030, Shimoogino, Atsugi, Kanagawa 243-0292 Japan

**Keywords:** Nucleotide excision repair, XPC, CSB, Environmental mutagen, Cytotoxicity, TK6

## Abstract

**Background:**

The human genome is constantly exposed to numerous environmental genotoxicants. To prevent the detrimental consequences induced by the expansion of damaged cells, cellular protective systems such as nucleotide excision repair (NER) exist and serve as a primary pathway for repairing the various helix-distorting DNA adducts induced by genotoxic agents. NER is further divided into two sub-pathways, namely, global genomic NER (GG-NER) and transcription-coupled NER (TC-NER). Both NER sub-pathways are reportedly involved in the damage response elicited by exposure to genotoxins. However, how disruption of these sub-pathways impacts the toxicity of different types of environmental mutagens in human cells is not well understood.

**Results:**

To evaluate the role of NER sub-pathways on the cytotoxic effects of mutagens, we disrupted *XPC* and *CSB* to selectively inactivate GG-NER and TC-NER, respectively, in human lymphoblastoid TK6 cells, a standard cell line used in genotoxicity studies. Using these cells, we then comparatively assessed their respective sensitivities to representative genotoxic agents, including ultraviolet C (UVC) light, benzo [a] pyrene (B(a)P), 2-amino-3,8-dimethylimidazo [4,5-f] quinoxaline (MeIQx), 2-amino-1-methyl-6-phenylimidazo [4,5-b] pyridine (PhIP), γ-ray, and 2-acetylaminofluorene (2-AAF). *CSB*^−/−^ cells exhibited a hyper-sensitivity to UVC, B(a)P, and MeIQx. On the other hand, *XPC*^−/−^ cells were highly sensitive to UVC, but not to B(a)P and MeIQx, compared with wild-type cells. In contrast with other genotoxins, the sensitivity of *XPC*^−/−^ cells against PhIP was significantly higher than *CSB*^−/−^ cells. The toxicity of γ-ray and 2-AAF was not enhanced by disruption of either *XPC* or *CSB* in the cells.

**Conclusions:**

Based on our findings, genetically modified TK6 cells appear to be a useful tool for elucidating the detailed roles of the various repair factors that exist to combat genotoxic agents, and should contribute to the improved risk assessment of environmental chemical contaminants.

## Introduction

Cellular DNA is continuously exposed to various environmental agents such as ultraviolet (UV) light from the sun, ionizing radiation, chemical compounds found in foods, and endogenous oxidative stress. This accumulation of DNA damage can be detrimental to cell viability because of the enhanced genomic instability, otherwise leading to cellular transformation and tumorigenesis. To maintain genomic integrity, mammalian cells possess multiple DNA repair pathways that recognize and resolve specific types of DNA damage. Among these, nucleotide excision repair (NER) is one of the most important repair pathways for counteracting DNA-damaging agents.

NER is involved in the removal of various helix-distorting DNA adducts that are induced by genotoxic agents [1]. In this process, NER can recognize almost an infinite variety of DNA adducts, including cyclobutane pyrimidine dimers induced by ultraviolet light (UV), covalent DNA adducts formed by carcinogens such as heterocyclic amines (HCAs) and polycyclic aromatic hydrocarbons (PAHs), DNA cross-links, and oxidative DNA damage from endogenous reactive oxygen species. Accordingly, the disruption of the NER machinery would be expected to enhance the toxic and mutagenic effects of genotoxic agents depending on the downstream pathways that result in cell death or carcinogenesis.

**Fig. 1:**
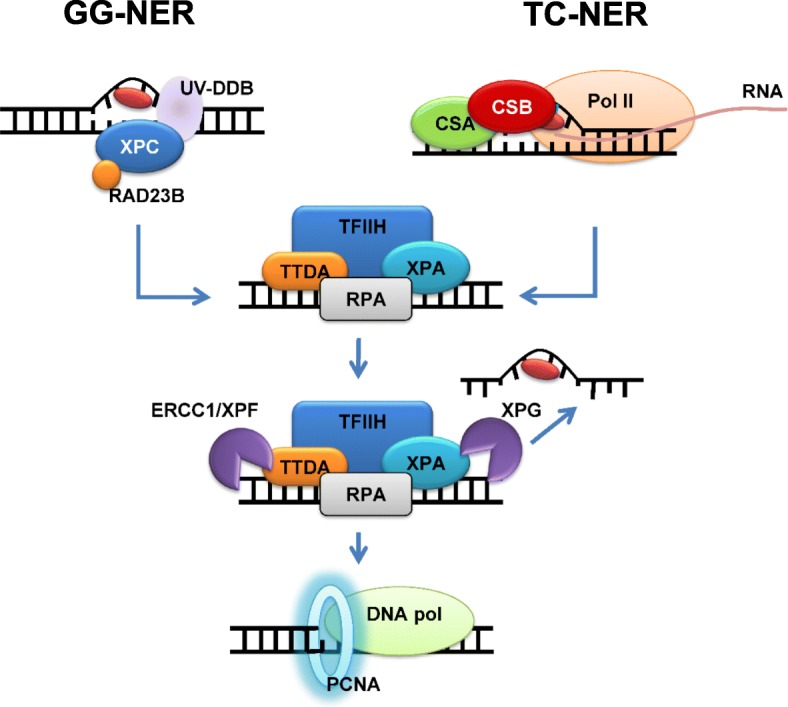
Schematic of nucleotide excision repair sub-pathways. In GG-NER, XPC and RAD23B complex with UV-damaged DNA-binding protein (UV-DDB) participate in the primary DNA damage recognition and recruitment of TFIIH complex. TC-NER is initiated by the stalling of RNA polymerase II (PolII) at the site of the DNA adduct. CSA and CSB are required for the removal of the stalled PolII and the assembly of the NER factors. Following the recruitment of the TFIIH complex to the DNA adduct site, both GG-NER and TC-NER share the same core NER factors, including ERCC1/XPF, XPG, and replication machinery [PCNA and DNA polymerase (DNA pol)], as depicted

NER is further divided into two sub-pathways, namely, global genomic NER (GG-NER) and transcription-coupled NER (TC-NER) (Fig. 1) [2]. GG-NER recognizes and excises DNA adducts from the entire genome, including transcribed and non-transcribed regions. On the other hand, TC-NER is responsible for removing transcription blocking-DNA adducts on actively transcribed genomic loci. In mammalian GG-NER, xeroderma pigmentosum group C (XPC) is involved in the essential role of the initial damage recognition step [3]. Indeed, pathogenic mutations in the gene encoding XPC result in a cancer-prone phenotype due to defective GG-NER [4]. In contrast, TC-NER is activated by stalling the elongation of RNA polymerase II at the site of the DNA adduct. Subsequently, the key factors, Cockayne syndrome protein A (CSA) and Cockayne syndrome protein B (CSB), initiate the onset of TC-NER. Following the recruitment of the transcription factor II H (TFIIH) complex to the damaged site, GG-NER and TC-NER engage the same protein components for incision of the DNA adduct, which is followed by gap-filling and ligation in order to complete the process. Because of the differential roles of these NER sub-pathways, the specific disruption of GG-NER or TC-NER allows for the elucidation of the underlying mechanisms of crosstalk between the cytotoxicity and the repair pathway of DNA adducts.

Both GG-NER and TC-NER factors, i.e., XPC and CSB, appear to regulate the role of the tumor suppressor p53. For example, CSB interacts with p53 and stimulates the ubiquitylation of the protein [5], with the loss of CSB resulting in the accumulation of p53 leading to an increased sensitivity of cells to cisplatin [5, 6]. The ATPase domain of CSB appears important for UV-induced apoptosis [7]. XPC also participates in the process of p53 degradation through the interaction with MDM2 [8]. In addition, XPC enhances DNA damage-induced apoptosis via downregulation of the anti-apoptotic isoform of caspase 2 [9]. Based on these findings, the NER key factors CSB and XPC have been suggested to be involved in the regulation of the equilibrium between the removal of highly damaged cells and the restoration of recoverable cells. Therefore, the pathogenic dysfunction of NER sub-pathways can lead to detrimental effects from exposure to environmental genotoxic agents. However, loss of each NER sub-pathway has revealed a distinct response upon exposure to a genotoxin [10, 11], and a phenotypic inconsistency of the NER deficiency has been reported between mice and humans [12]. Thus, the impact of NER sub-pathways on the cytotoxic response to different mutagens in human cells remains to be elucidated.

To better understand the role of NER sub-pathways on the cytotoxicity of genotoxic agents, we developed human lymphoblastoid TK6-isogenic cells lacking the function of XPC or CSB to specifically disrupt GG-NER or TC-NER, respectively. Human TK6 is a standard and widely used cell line for in vitro genotoxicity testing [13]. Using these cells, we comparatively evaluated the cellular sensitivities to genotoxic agents, including ultraviolet C (UVC), benzo [a] pyrene (B(a)P) as PAHs, 2-amino-3,8-dimethylimidazo [4,5-f] quinoxaline (MeIQx) and 2-amino-1-methyl-6-phenylimidazo [4,5-b] pyridine (PhIP) as HCAs, γ-rays that induce DNA strand breaks, and 2-acetylaminofluorene (2-AAF). Our results demonstrated that GG-NER- and TC-NER-deficient TK6 cells are a good tool for clarifying the mechanisms of DNA damage in the cellular response to genotoxic agents.

## Materials and methods

### Cell culture

The human lymphoblastoid cell line TSCER122, an isogenic derivative of TK6, was used in this study [14]. Cells were cultured in RPMI-1640 medium (Nacalai Tesque) with 10% horse serum (Nichirei Biosciences, Inc.), 100 U/mL penicillin, 100 μg/mL streptomycin, and 200 μg/mL sodium pyruvate at 37 °C, 5% CO_2_, and 100% humidity.

### Generation of *XPC*^−/−^ and *CSB*^−/−^ cells

**Fig. 2:**
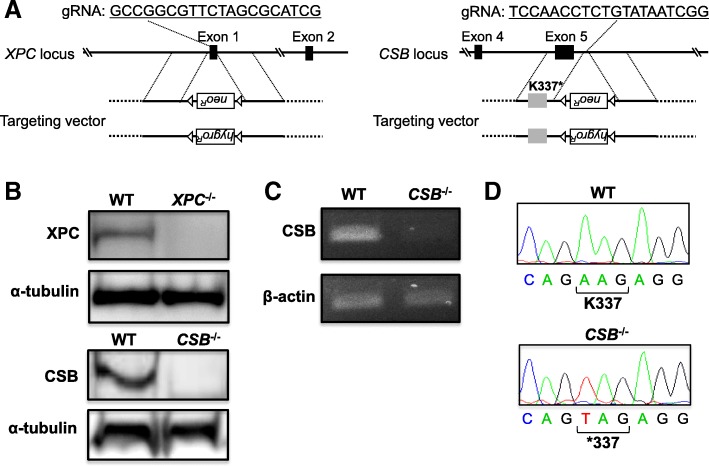
Generation of *XPC*^−/−^ and *CSB*^−/−^ cells. **a** Schematic representation of the targeted disruption of *XPC* and *CSB.* The target sequence of CRISPR/Cas9 and the targeting vector containing a neomycin-resistance (*neo*^R^) or hygromycin-resistance (*hygro*^R^) marker cassette in the opposite direction are shown. The black boxes and triangles represent the exons and *loxP* sequences, respectively. **b** Western blot analysis for the XPC and CSB proteins. Whole cell extracts of WT, *XPC*^−/−^, and *CSB*^−/−^ were loaded onto a 10% SDS-polyacrylamide gel. α-Tubulin served as a loading control. **c** RT-PCR analysis for CSB mRNA. The same amounts of total RNA extracted from each cell were used. β-Actin served as an internal control. **d** The sequence of CSB cDNA generated by RT-PCR. The sequences around codon 337 are shown

To generate the *XPC*^−/−^ and *CSB*^−/−^ cells, we designed guide RNA (gRNA) targets for CRISPR/Cas9 genome editing in combination with gene targeting constructs. CRISPR-target sequences are depicted in Fig. 2A. gRNAs were inserted into the *Bbs*I site of the pX330 vector as described previously [15].

For disruption of *XPC*, DNA fragments were obtained by PCR from TK6 genomic DNA using the following primers: 5′-ATTCGAGCTCGGTACGAGGCTTCCTCTGATCATCTAACT-3′ and 5′-GCTCGAGGGGGGGCCAGGCCTAGTCACGCCCCTAAAG-3′ for the 5′-arm, 5′-GGGAAGCTTGTCGACGAACTGCGCAGCCAGAAATC-3′ and 5′-GCTTGCATGCCTGCAGGTTCACTCTAGGCAGAAGGAAC-3′ for the 3′-arm. The DT-A-pA/loxP/PGK-*neo*^*R*^-pA/loxP vector was provided by the Laboratory for Animal Resources and Genetic Engineering, Center for Developmental Biology, Institute of Physical and Chemical Research, Kobe, Japan. The 5′- and 3′-arms were introduced into the *Apa*I and *Afl*II sites of the DT-A-pA/loxP/PGK-*neo*^*R*^-pA/loxP or DT-A-pA/loxP/PGK-*hygro*^*R*^-pA/loxP vectors, respectively, by using a GeneArt Seamless cloning kit (Thermo Fisher Scientific). The resulting targeting vectors were named pDT-A-XPC-*neo*^*R*^ and pDT-A-XPC-*hygro*^*R*^. The vectors pX330-gRNA (6 μg), pDT-A-XPC-*neo*^*R*^ (2 μg), and pDT-A-XPC-*hygro*^*R*^ (2 μg) were then transfected into cells by the Neon transfection system (Thermo Fisher Scientific). After 48 h incubation, cells were seeded into 96-microwell plates in the presence of G-418 (1 mg/mL) and hygromycin (0.625 mg/mL). Drug-resistant cell colonies were picked 10 days after transfection and subjected to genomic PCR using the following primers: 5′-CTACTGCTACCTAAGCCTCTGTCTG-3′ and 5′-CGCCTTCTATCGCCTTCTTGACGAGTTCTT-3′ for a targeted allele with neomycin-resistance cassette or 5′-CTACTGCTACCTAAGCCTCTGTCTG-3′ and 5′-TGACGGCAATTTCGATGATGCAGCTTGG-3′ for a targeted allele with hygromycin-resistance cassette. For disruption of *CSB*, DNA fragments were obtained by PCR using the primers 5′-ATTCGAGCTCGGTACGATGCTATAATTTTATTCTGTCCTTC-3′ and 5′-GCTCGAGGGGGGGCCAACCTCTGTATAATCGGGGG-3′ for the 5′-arm, 5′- GGGAAGCTTGTCGACGCAGCCTTAACCTGCTAGAAGC-3′ and 5′-GCTTGCATGCCTGCACTAGAATGTGAGTGCCGCAACT-3′ for the 3′-arm. The mutation of AAG to TAG at codon 337, which mimics the CSB patient mutation (Lys337 → stop, K337*) [16], was introduced into the 5′-arm by PCR-mediated site-directed mutagenesis. The 5′- and 3′-arms were then introduced into the *Apa*I and *Afl*II sites of the DT-A-pA/loxP/PGK-*neo*^*R*^-pA/loxP or DT-A-pA/loxP/PGK-*hygro*^*R*^-pA/loxP vectors, respectively, as described above. The resulting targeting vectors were named pDT-A-CSB-*neo*^*R*^ and pDT-A-CSB-*hygro*^*R*^. The vectors pX330-gRNA (6 μg), pDT-A-CSB-*neo*^*R*^ (2 μg), and pDT-A-CSB-*hygro*^*R*^ (2 μg) were then transfected into the cells by the Neon transfection system (Thermo Fisher Scientific). After 48 h incubation, cells were seeded into 96-microwell plates in the presence of G-418 (1 mg/mL) and hygromycin (0.625 mg/mL). Drug-resistant cell colonies were picked 10 days after transfection and subjected to genomic PCR using the following primers: 5′-CCTATCTTTGGATGGCAGAGAGTAT-3′ and 5′-CGCCTTCTATCGCCTTCTTGACGAGTTCTT-3′ for a targeted allele with neomycin-resistance cassette or 5′-CCTATCTTTGGATGGCAGAGAGTAT-3′ and 5′-TGACGGCAATTTCGATGATGCAGCTTGG-3′ for a targeted allele with hygromycin-resistance cassette.

### RT-PCR

Total RNA was extracted from the cells using an RNeasy mini kit (Qiagen). The RNA was then reverse transcribed using SuperScript III Reverse Transcriptase (Thermo Fisher Scientific). Synthesized cDNA was subjected to PCR using the following primers: 5′-GGAGGAGCAGAGGTGAAAATTGAAC-3′ and 5′-CTCCTCTGTGGGGAAATACTCAGA-3′ for CSB or 5′-GCTCGTCGTCGACAACGGCTC-3′ and 5′-CAAACATGATCTGGGTCATCTTCTC-3′ for β-actin. The introduction of the expected mutation into the targeted alleles of the *CSB*^−/−^ cells was confirmed by DNA sequencing.

### Western blotting

Total cell extracts were fractioned on 10% SDS-polyacrylamide gels and subsequently transferred to PVDF membranes. Membranes were blocked with 1% BSA for XPC or 5% skim milk for CSB and α-tubulin. To detect XPC, CSB, and α-tubulin, membranes were incubated with a 1:500 dilution of anti-XPC monoclonal antibody (ab6264, Abcam), 1:200 dilution of anti-CSB polyclonal antibody (H-300, Santa Cruz), or 1:10000 dilution of anti-α-tubulin monoclonal antibody (ab7291, Abcam) overnight in Can Get Signal Solution 1 (Toyobo). After washing with phosphate-buffered saline containing 0.05% Tween 20, membranes were incubated with a 1:2500 dilution of anti-rabbit IgG conjugated to horseradish peroxidase (GE Healthcare). The chemiluminescent signal was detected using the ECL Prime Western blotting detection reagent (GE Healthcare).

### UV irradiation

Cells were washed and resuspended with RPMI-1640 without phenol red. Cells (2.5 × 10^6^) in 5 mL medium were exposed to UVC (0, 0.5, 1.0, 1.5, and 2.0 J/m^2^) in 10-cm Petri dishes. Irradiated cells were then seeded into 96-microwell plates at 8 cells/mL (1.6 cells/well) to determine cell survival. Colonies in 96-microwell plates were scored after 14 days.

### Chemical treatment

MeIQx and PhIP were from Wako Pure Chemical Industries Ltd. (Osaka, Japan). B(a)P was purchased from *Sigma-*Aldrich (St. Louis, MO). 2-AAF was obtained from Tokyo Chemical Industry Co., Ltd. (Tokyo, Japan). All chemicals were dissolved in dimethyl sulfoxide. For cellular exposure to the chemicals, 10 mL of cell suspensions at a concentration of 5 × 10^6^ cells was treated with chemicals in the presence of rat liver S9 mix (Oriental Yeast Co., Ltd. and BoZo Research Center Inc.) at a concentration of 4.5% for metabolic activation. After 3 h of treatment at 37 °C with gentle shaking, cells were washed with RPMI-1640 medium twice and then seeded into 96-microwell plates as described in the previous section in order to determine cell survival.

### γ-Ray irradiation

γ-Ray irradiation was performed using a Gammacell 40 Exactor (MDS Nordion, Canada). Irradiated cells were seeded into 96-microwell plates as described in the previous section in order to determine cell survival.

### Statistical analysis

Cell survival was compared between WT, *XPC*^−/−^, and *CSB*^−/−^ cells at the same dose concentrations using Tukey’s multiple comparison test. The level of statistical significance was set at *P* < 0.05.

## Results and discussion

### Generation of *XPC*^−/−^ and *CSB*^−/−^ cells

To selectively inactivate the NER sub-pathways, *XPC* and *CSB* were chosen as the target genes for disruption of GG-NER and TC-NER, respectively. We disrupted *XPC* using the gene targeting methodology depicted in Fig. 2a. Similarly, we inserted the K337* mutation in exon 5 of *CSB* using the same targeting technique. The resulting *XPC*^−/−^ and *CSB*^−/−^ cells did not express XPC or the full-length CSB protein, respectively (Fig. 2b). As shown in Fig. 2c, decreased expression of *CSB* was observed by the introduction of the K337* mutations into both alleles of the *CSB* loci, which may be because of the presence of the nonsense-mediated mRNA decay system in the cells [17]. DNA sequencing of the cDNA revealed that the mutated mRNA was expressed in the *CSB*^−/−^ cells (Fig. 2d). Using these isogenic cell lines, the effect of each NER sub-pathway on the cytotoxicity of various genotoxic agents was then assessed. It should be noted that the treatment of cell lines with S9 mix for metabolic activation did not have a significant effect on the growth and number of colonies formed in the following assay.

### Ultraviolet light

**Fig. 3:**
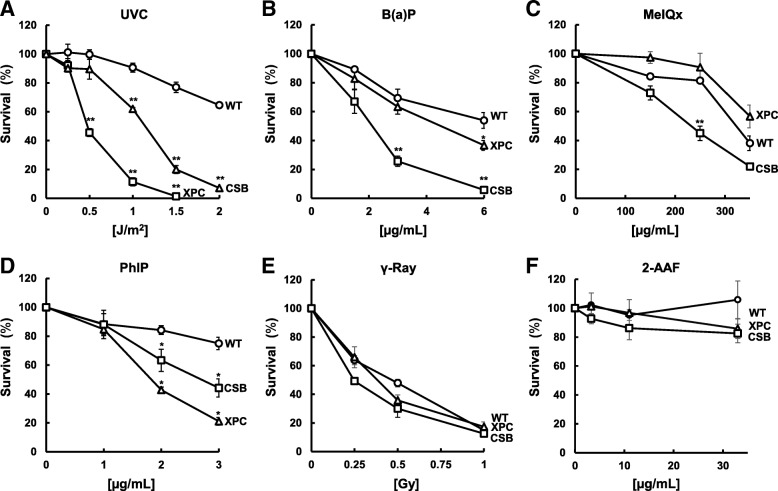
Cytotoxicity of genotoxic agents in XPC- and CSB-deficient human TK6 cells. Survival of WT (circles), *XPC*^−/−^ (triangles), and *CSB*^−/−^ (squares) cells was shown after exposure to UVC (**a**), B(a) P (**b**), MeIQx (**c**), PhIP (**d**), γ-ray (**e**), and 2-AAF (**f**). Values presented are means ± SEM of 2–4 independent experiments. Experiments were performed as described in Materials and Methods. Significant differences are indicated by asterisks (**p < 0.01, *p < 0.05)

To confirm the contribution of GG-NER and TC-NER to cell survival after UV-exposure, we compared the sensitivity of the WT, *XPC*^−/−^, and *CSB*^−/−^ cell lines to UVC irradiation. As shown in Fig. 3a, both *XPC*^−/−^ and *CSB*^−/−^ cells were considered hyper-sensitive compared with WT. Notably, *XPC*^−/−^ cells displayed a more severe phenotype against UV-induced cytotoxicity than that observed with *CSB*^−/−^ cells. Previous studies have suggested the differential UV-induced sensitivity of *Xpc*^−/−^ and *Csb*^−/−^ mouse cells depending on the cell-type; *Csb*^−/−^ mouse embryonic fibroblasts (MEFs) and keratinocytes are highly sensitive to UV irradiation compared with *Xpc*^−/−^ cells, but *Xpc*^−/−^ embryonic stem cells are more susceptible to UV exposure than *Csb*^−/−^ cells [18, 19]. In the former case, the enhanced sensitivity of *Csb*^−/−^ cells to UV irradiation is likely due to the increased level of apoptotic response in *Csb*^−/−^ cells [18, 19]. In the latter case, deficiency of *Csb* results in higher UV-induced mutation rate than *Xpc* deficiency [18], resulting in proliferation of damaged cells. Thus, among the NER sub-pathways, not only the disruption of CSB, but also the loss of XPC is more detrimental to cellular survival upon exposure to UV in human TK6 cells. The severe sensitivity of the cells to UVC can be because of the role of these NER factors to selectively eliminate highly damaged abnormal cells via apoptosis in humans [9].

### B(a)P

B(a)P is a potent PAH carcinogen present in tobacco smoke and is formed during the incomplete combustion of organic materials [20]. B(a)P is metabolized to the reactive intermediate benzo [*a*]pyrene-7,8-dihydrodiol-9,10-epoxide (BPDE), which forms bulky DNA adducts in cells [21]. In order to define B(a)P-induced cytotoxicity, cell survival after exposure to the chemical was determined. Cellular sensitivity to B(a)P was dramatically enhanced by deficiency of *CSB* [significant at 3 and 6 μg/mL B(a) P (*p* < 0.01)] (Fig. 3b). On the other hand, the sensitivity of *XPC*^−/−^ cells was only slightly higher than WT, which was significant at 6 μg/mL B(a)P (*p* < 0.05). These results indicated that *XPC*^−/−^ cells were more resistant to B(a)P compared with *CSB*^−/−^ cells. It has been shown that exposure of BPDE to human cells induces the upregulation of NER genes, including *XPC*, *XPF*, *XPG*, and *DDB2*, in a p53-dependent manner [22]. However, the expression of *CSB* is not enhanced upon exposure to BPDE [22]. According to these findings and our results, XPC appears to have a major role in eliminating highly damaged cells via DNA damage-induced apoptosis in the absence of TC-NER, as suggested by the previous reports showing that the increased level of apoptotic response in *Csb*^−/−^ cells, but not in *Xpc*^−/−^ mouse cells in response to genotoxic stress [18, 19].

### MeIQx

MeIQx is an HCA that is present in cooked meat and fish. We examined the roles of the GG-NER and TC-NER factors in the cellular tolerance to the cytotoxicity of MeIQx. The disruption of *CSB* significantly sensitized cells to MeIQx at 250 μg/mL (*p* < 0.01) (Fig. 3c). On the other hand, *XPC*^−/−^ cells were somewhat less sensitive compared with WT, although these differences were not statistically significant at all doses (150, 250, and 250 μg/mL). This observation can be explained by the amount of oxidative DNA damage induced by MeIQx. Upon the exposure of rats to MeIQx at low doses, the formation of bulky MeIQx-DNA adducts has been observed [23]. However, the increasing dosage of MeIQx significantly elevated the level of oxidative DNA damage in tissues in the form of 7,8-dihydro-8-oxoguanine (8-oxoG). It should be noted that cells derived from CSB patients have been shown to be hyper-sensitive to oxidative DNA damage [24]. Evidences for the involvement of CSB in the repair of oxidized base damages have also been reported [25–27]. Therefore, enhanced sensitivity in *CSB*^−/−^ cells and the loss of sensitivity in *XPC*^−/−^ cells against MeIQx are likely due to both the CSB-mediated repair of oxidative DNA damage and XPC-induced apoptosis in cells. Collectively, the inactivation of TC-NER, but not GG-NER, served to enhance the cytotoxicity of MeIQx in human cells.

### PhIP

PhIP, another HCA and well-known food carcinogen, causes bulky DNA adducts at the C8 position of guanine [28]. Contrary with the other genotoxic agents assessed, *XPC*^−/−^ cells displayed the highest sensitivity among the three cell lines against PhIP treatment [significant at 2 and 3 μg/mL PhIP (*p* < 0.05)] (Fig. 3d). *CSB*^−/−^ cells revealed an intermediate sensitivity between that of WT and *XPC*^−/−^. Our results are in contrast to a previous study that found no differences in the toxicity of PhIP treatment with *Xpc* knock-out mice in comparison with wild-type [29]. This discrepancy is possibly due to the importance of GG-NER in humans compared with rodents as reported previously [30]. The exposure of TK6 cells to PhIP induces p53 activation, which is true for other genotoxic agents that form bulky DNA adducts [31]. In the case of PhIP, XPC may play a suppressive role in cell death via a mechanism that includes the degradation of p53 [8]. Hence, our results indicate that the loss of GG-NER is critical to the cytotoxic events following the accumulation of PhIP-DNA adducts. It has been reported that PhIP induces not only bulky DNA adducts but also DNA double-strand breaks (DSBs) [32], arising the possibility that the enhanced toxicity of PhIP in NER-deficient cells may be due to the formation of DSBs upon PhIP treatment. Thus, we further investigated cellular survival following exposure to γ-rays that induce DSBs.

### γ-Rays

It has been reported that the disruption of the *Csb* gene sensitizes MEFs to ionizing radiation [33]. A possible involvement of XPC in DSB repair has also been suggested [34]. We therefore assessed whether the loss of XPC and CSB contributes to the cytotoxicity of γ-ray exposure in human lymphoblastoid cells. Cell viability decreased and was dependent on the increasing dosage of γ-rays in WT, *XPC*^−/−^, and *CSB*^−/−^ cells (Fig. 3e). Although the sensitivity of *CSB*^−/−^ cells against γ-rays was slightly higher than WT cells, there were no significant differences observed between the cell lines. While CSB and XPC do play some roles in DSB repair, the absence of these NER factors did not have a significant impact on the cytotoxicity of γ-rays in TK6 cells.

### 2-AAF

2-AAF is a mutagenic derivative of fluorene capable of forming bulky DNA adducts [35]. We next examined whether the disruption of *XPC* or *CSB* alters 2-AAF-induced cytotoxicity. As shown in Fig. 3f, exposure to 2-AAF (3.3, 11, and 33 μg/ml) did not sensitize *XPC*^−/−^ and *CSB*^−/−^ cells. Further increasing dose of 2-AAF resulted in the precipitation in the medium during the chemical treatment period, which was not a reliable assay condition.

## Conclusions

In this study, we assessed the effect of GG-NER and TC-NER inactivation on the toxicity induced by several environmental mutagens that form bulky DNA adducts. Remarkably, the NER-deficient cells revealed differential sensitivity from the exposure to genotoxic agents, and this was dependent on the DNA adducts formed and the following repair and/or apoptotic mechanisms to be induced. On the basis of these results, we propose that the GG-NER- and TC-NER-deficient cells established in this study will be useful for investigating the mechanisms involved in the individual stages of chemical toxicity, in combination with standard genotoxicity tests. For example, the correlation between symptoms remains unclear, e.g., neurodegeneration, in CS/XP patients and chronic exposure to environmental chemical contaminants in daily life. Accordingly, these cells provide a tool for assessing the risks of exposure to drugs and chemicals that are associated with NER deficiency. Furthermore, these TK6 isogenic cells enable the tracing of the fate of site-specific DNA damage in the defined locus of the thymidine kinase gene [14, 36, 37]. These methodologies may thus shed light on the detailed protective roles of NER factors against genotoxic agents and contribute to the improved risk assessment of chemicals.

## Data Availability

Not applicable.

## Abbreviations

2-AAF: 2-acetylaminofluorene
8-oxoG: 7,8-dihydro-8-oxoguanine
B(a)P: Benzo [a]pyrene
BPDE: Benzo [*a*]pyrene-7,8-dihydrodiol-9,10-epoxide
CPD: Cyclobutane pyrimidine dimers
CSA: Cockayne syndrome protein A
CSB: Cockayne syndrome protein B
DSBs: DNA double-strand breaks
GG-NER: Global genomic NER
HCAs: Heterocyclic amines
MEFs: Embryonic fibroblasts
MeIQx: 2-amino-3,8-dimethylimidazo [4,5-f]quinoxaline
NER: Nucleotide excision repair
PAHs: Polycyclic aromatic hydrocarbons
PhIP: 2-amino-1-methyl-6-phenylimidazo [4,5-b]pyridine
TC-NER: Transcription-coupled NER
TFIIH: Transcription factor II H
UV: Ultra violet
XPC: Xeroderma pigmentosum group C
